# Effects of CRISPR-Cas9-mediated *FOXP3* knockout on CAR T cell potency

**DOI:** 10.1016/j.omtm.2025.101570

**Published:** 2025-08-21

**Authors:** Lena Peter, Martí Farrera-Sal, Ferhat Ali Yaman, Nils Henrik Dempewolf, Samira Picht, Sarah Schulenberg, Jonas Kath, Frederik Hamm, Frederik Heinrich, Dimitrios L. Wagner, Mir-Farzin Mashreghi, Annette Künkele, Petra Reinke, Julia K. Polánsky, Michael Schmueck-Henneresse

**Affiliations:** 1 Berlin Institute of Health (BIH) at Charité – Universitätsmedizin Berlin, BIH Center for Regenerative Therapies (BCRT), Augustenburger Platz 1, 13353 Berlin, Germany; 2 Berlin Center for Advanced Therapies (BeCAT), Charité – Universitätsmedizin Berlin, 13353 Berlin, Germany; 3 German Rheumatism Research Center, Institute of Leibniz-Gemeinschaft, Charitéplatz 1, 10117 Berlin, Germany; 4 Center for Cell and Gene Therapy, Department of Molecular and Cellular Biology, Baylor College of Medicine, Houston, TX 77030, USA; 5 Department of Molecular and Cellular Biology, Baylor College of Medicine, Houston, TX 77030, USA; 6 Dan L Duncan Comprehensive Cancer Center, Baylor College of Medicine, Houston, TX 77030 , USA; 7 Department of Pediatric Oncology and Hematology, Charité - Universitätsmedizin Berlin, Berlin, Germany; 8 The German Cancer Consortium (DKTK), Partner Site Berlin, Berlin, Germany

**Keywords:** FOXP3, CRISPR-Cas9, CAR T cells, T cell exhaustion, effector T cells, immunotherapy, gene editing, cytotoxicity, CAR T cell potency, immunomodulation

## Abstract

Persistent antigen stimulation and inflammatory environments drive exhaustion, senescence, and activation-induced cell death, impairing both endogenous and therapeutic T cells. Understanding the mechanisms underlying T cell dysfunction is critical for improving immunotherapies. While the transcription factor forkhead box protein P3 (FOXP3) is primarily known for its role in regulatory T cell development and maintenance, recent studies suggest it may also influence effector T cell function. However, its impact on therapeutic T cells, including CAR T cells, remains poorly defined. Here, we used non-viral CRISPR-Cas9 editing to knockout *FOXP3* in CD19-directed CAR T cell products (TCPs) generated via lentiviral transduction. FOXP3 expression was upregulated at both the protein and RNA level following CAR stimulation. Compared to unmodified CAR TCPs, *FOXP3*-KO CAR TCPs showed comparable exhaustion profiles but enhanced cytokine production and prolonged cytotoxic function across repeated antigen challenges. These findings identify FOXP3 as a context-dependent modulator of CAR T cell function and suggest that its disruption may enhance therapeutic potency without exacerbating exhaustion. FOXP3 targeting may represent a complementary strategy to improve the functional resilience of CAR T cell therapies in cancer or autoimmune disease.

## Graphical abstract

**Figure:**
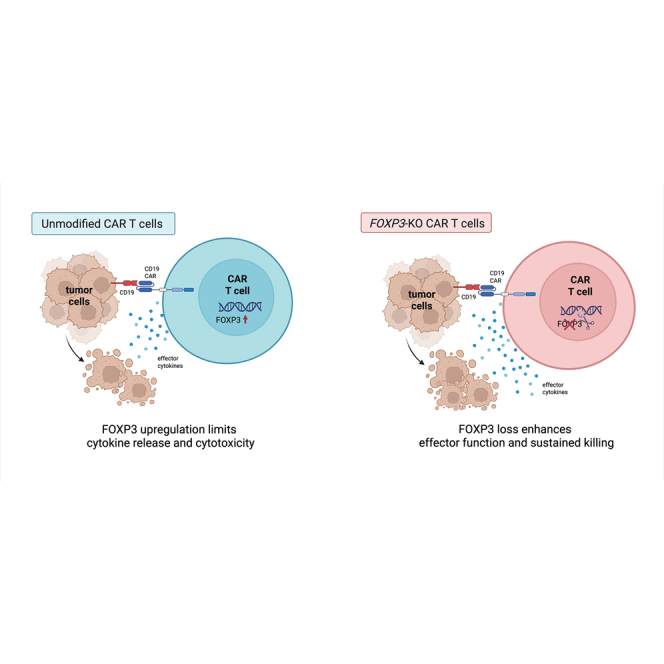

## Teaser

Peter and colleagues demonstrate that FOXP3, traditionally known for its role in regulatory T cells, limits effector function in CAR T cells. Using CRISPR-Cas9 knockout, they reveal FOXP3 as a potential modulator of therapeutic T cell activity, offering a strategy to improve CAR T cell efficacy in disease settings.

## Introduction

T cells are a fundamental component of the adaptive immune system, playing a central role in defending against pathogens and malignant cells. Among them, effector T cells (Teff) eliminate infected or transformed cells upon antigen recognition through their T cell receptors (TCRs), a process critical for cell-mediated immunity and the foundation for many immunotherapeutic strategies.^1^ However, in chronic infection and cancer, persistent antigen exposure and inflammatory conditions often lead to T cell dysfunction, characterized by progressive exhaustion.^2^ Exhausted T cells exhibit transcriptional, epigenetic, and metabolic alterations, leading to diminished effector functions, impaired proliferation, and upregulation of inhibitory receptors.^2^ T cell exhaustion presents a major challenge in chimeric antigen receptor (CAR) T cell therapy, an approach that has shown remarkable success in treating blood cancers.^3^^,^^4^ However, the long-term efficacy of CAR T cells is hindered by repeated antigen stimulation, which drives exhaustion, and by the immunosuppressive tumor microenvironment, which undermines their persistence and function.^4^^,^^5^^,^^6^ Recent evidence suggests that T cell functionality may be influenced by forkhead box protein P3 (FOXP3), the master transcription factor of regulatory T cells (Tregs), which suppress immune responses through inhibitory cytokine production, metabolic disruption, and suppression of antigen-presenting cells.^7^^,^^8^^,^^9^ While FOXP3 is constitutively expressed in Tregs, human conventional Teff can transiently upregulate FOXP3 upon activation,^10^^,^^11^^,^^12^^,^^13^^,^^14^ suggesting it may act as a negative regulator of Teff function. Indeed, studies indicate that FOXP3 expression correlates with reduced cytokine production and cytotoxicity,^11^^,^^13^ and recent findings suggest that TCR-induced FOXP3 expression in CD8^+^ T cells impairs their anti-tumor activity.^13^^,^^14^ Although the mechanisms underlying FOXP3-driven dysfunction remain to be fully defined, its association with impaired effector function prompted us to assess the impact of *FOXP3* deficiency on CAR T cell potency and exhaustion profiles. In contrast to CAR-Treg studies that have leveraged FOXP3 overexpression to stabilize regulatory function, our study interrogates the consequences of FOXP3 loss in effector CAR T cells. To test this, we used non-viral CRISPR-Cas9 gene editing to disrupt *FOXP3* in CAR T cell products (TCPs) targeting CD19, generated via lentiviral transduction. Unmodified CAR TCPs, with intact FOXP3 gene, were generated in parallel and served as control. *FOXP3*-KO and unmodified CAR TCPs were expanded and compared under conditions of repetitive antigen exposure. Upon CAR stimulation, FOXP3 expression increased in unmodified CAR TCPs at both the mRNA and protein levels. While *FOXP3*-KO and unmodified CAR TCPs exhibited similar exhaustion profiles, *FOXP3*-KO CAR TCPs showed a trend toward enhanced effector function, including increased cytokine production and prolonged cytotoxic activity. These findings suggest that FOXP3 expression may limit CAR T cell function, providing a foundation for exploring strategies to improve CAR T cell persistence and potency through *FOXP3* modulation.

## Results

### Lentiviral CAR delivery and CRISPR-Cas9-mediated *FOXP3* knockout in T cells

**Figure 1:**
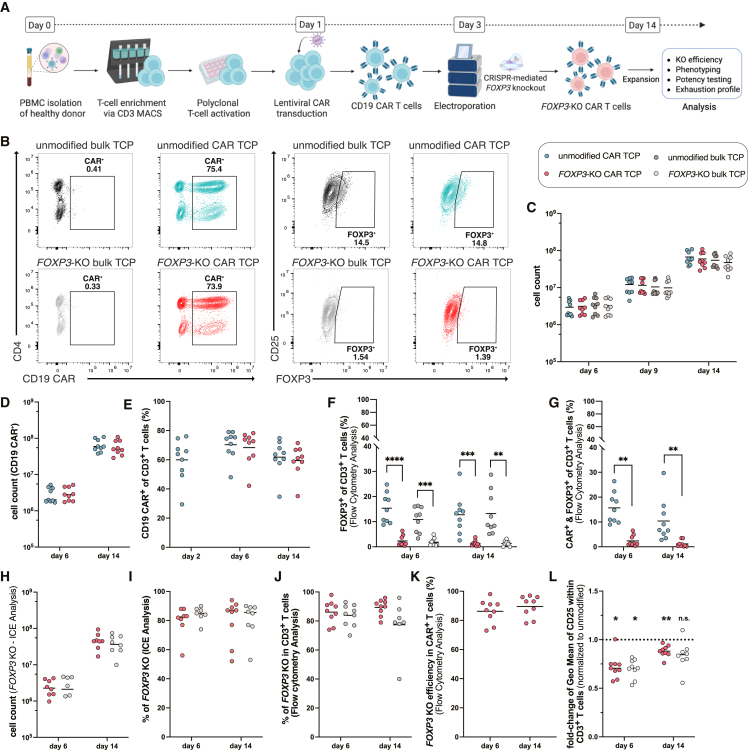
Generation and characterization of *FOXP3*-KO CAR T cell products Data represent mean from *n* = 9 independent donors (one dot in each graph represent one independent donor), unless stated otherwise. ∗*p* < 0.05, ∗∗*p* < 0.01, ∗∗∗*p* < 0.001, ∗∗∗∗*p* < 0.0001. (A) Schematic representation of the experimental workflow. PBMCs were isolated from healthy donors; CD3^+^ T cells were enriched, activated with CD3/CD28 antibodies, transduced with lentiviral CD19 CAR construct on day 1, and subjected to CRISPR-Cas9-mediated *FOXP3* knockout on day 3. Created with BioRender.com. (B) Representative flow cytometry plots showing CAR expression (left) and FOXP3 levels (right) in the four T cell product (TCP) groups. (C) Expansion kinetics of unmodified and *FOXP3*-KO T cells in both CAR and bulk TCPs from days 6–14 post-CAR delivery. (D) Expansion of CAR^+^ T cells within respective TCPs, calculated based on flow cytometry data. (E) Flow cytometric analysis of CAR transduction efficiency on days 2, 6, and 14 post-CAR delivery within respective TCPs. (F) Flow cytometric analysis of FOXP3 expression levels in CAR and bulk TCPs at day 6 and 14 post-CAR delivery. (G) Flow cytometric analysis of FOXP3 expression levels in CAR^+^ TCPs at day 6 and 14 post-CAR delivery. (H) Expansion rates of T cells carrying *FOXP3* knockout, according to sanger sequencing and ICE analysis, within *FOXP3*-KO CAR and bulk TCPs. *n* = 8 for day 6 *FOXP3*-KO CAR TCPs and *n* = 6 for day 6 *FOXP3*-KO bulk TCPs. (I) Calculation of *FOXP3* knockout efficiency by sanger sequencing following ICE analysis on days 6 and 14. *n* = 8 for day 6 *FOXP3*-KO CAR TCPs and *n* = 6 for day 6 *FOXP3*-KO bulk TCPs. (J) *FOXP3* knockout efficiency on protein level in *FOXP3*-KO CAR and bulk TCPs on days 6 and 14 post-CAR delivery, calculated based on flow cytometry data. (K) *FOXP3* knockout efficiency on protein level in CAR^+^ T cells of *FOXP3*-KO CAR TCPs on days 6 and 14 post-CAR delivery, calculated based on flow cytometry data. (L) Flow cytometric analysis of CD25 (IL-2Rα) expression in *FOXP3*-KO CAR and bulk TCPs normalized to unmodified TCPs on days 6 and 14 post-CAR delivery.

We established a protocol combining lentiviral CAR delivery and non-viral CRISPR-Cas9-mediated *FOXP3* knockout (Figure 1A). Starting with peripheral blood mononuclear cell (PBMC) isolation from healthy donors and CD3 MACS enrichment, T cells were activated using plate-bound CD3/CD28 antibodies. The CAR construct was introduced via lentiviral transduction on day 1, followed by CRISPR-Cas9-mediated non-viral *FOXP3* knockout by electroporating ribonucleoprotein (RNP) complexes on day 3. The resulting TCPs were expanded for functional analyses, with four experimental groups: unmodified CAR TCP, *FOXP3*-KO CAR TCP, unmodified bulk TCP (lacking CAR), and *FOXP3*-KO bulk TCP (lacking CAR). Flow cytometric analysis revealed comparable CAR transduction efficiency between unmodified and *FOXP3*-KO CAR TCPs from day 6 to day 14 post-CAR delivery (Figures 1B, left panel and 1E). FOXP3 expression was significantly reduced in both CAR and bulk *FOXP3*-KO TCPs compared to their unmodified counterparts, confirming successful gene editing (Figures 1B, right panel, 1F, and 1G). No substantial differences in overall expansion rates were observed across all TCP groups between days 6, 9, and 14 post-CAR delivery (Figure 1C), a pattern consistent when examining CAR-positive cells within respective products (Figure 1D) or *FOXP3*-KO T cells of both CAR and bulk TCPs (Figure 1H).

*FOXP3* knockout efficiency was validated through multiple approaches. Targeted Sanger sequencing and interference of CRISPR edits (ICE) analysis^15^^,^^16^ confirmed genetic disruption at the DNA level (Figure 1I); flow cytometry analysis demonstrated corresponding protein reduction (Figures 1B, right panel, 1J, and 1K). Further validation in T cell subsets confirmed reduced FOXP3 protein expression in both bulk and CAR-expressing CD4^+^ T cells (Figures S1A and S1B) and CD8^+^ T cells (Figures S1C and S1D). Notably, decreased CD25 (interleukin-2 receptor alpha [IL-2Rα]) expression was observed in *FOXP3*-KO T cells compared to unmodified controls in both CAR and bulk TCPs at days 6 and 14 post-CAR delivery (Figure 1L). Next, we assessed whether the *FOXP3*-KO would interfere with the epigenetic imprinting of the T cell phenotype. Epigenetic profiling based on DNA methylation profiles using the Infinium MethylationEPIC Array revealed no joint clustering of *FOXP3*-KO TCPs in a principal-component analysis (Figure S1E). Moreover, no statistically significant differentially methylated positions (DMPs) were identified between *FOXP3*-KO and unmodified TCPs (data not shown). This indicated that there is no interruption of the T cell phenotype on the epigenetic levels resulting from the *FOXP3*-KO treatment. When focusing on the target gene *FOXP3* itself, our analysis also did not reveal significant changes in the methylation level of any methylation site (CpGs motifs) in the *FOXP3* locus included in the EPIC Array (Figure S1F).

### *FOXP3* knockout alters the phenotypic composition of CAR TCPs

**Figure 2:**
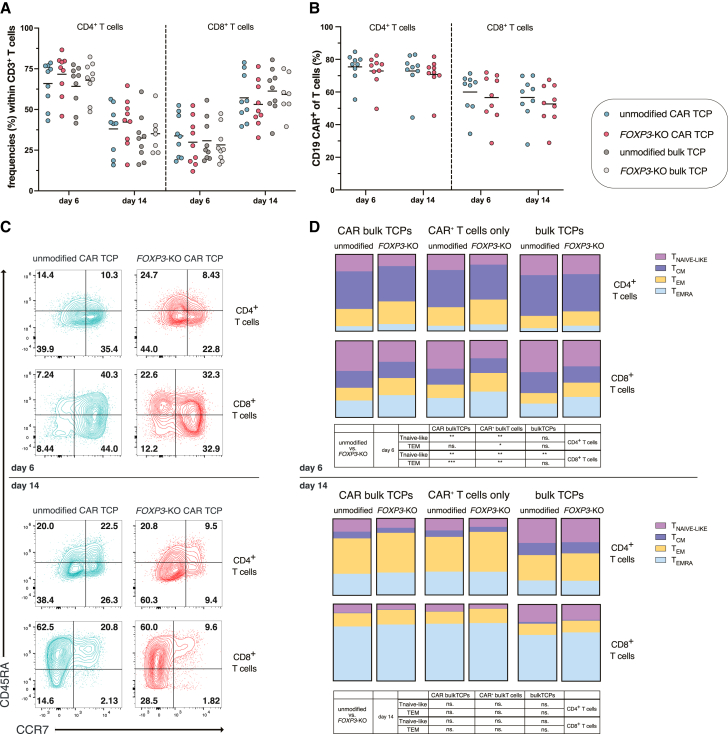
Impact of *FOXP3* knockout on T cell subset distribution and memory phenotype Data represent mean from *n* = 9 independent donors, (one dot in each graph represents one independent donor). ∗*p* < 0.05, ∗∗*p* < 0.01, ∗∗∗*p* < 0.001. (A) Flow cytometric analysis of CD4^+^ and CD8^+^ T cell frequencies in unmodified and *FOXP3*-KO T cells of both CAR and bulk TCPs on days 6 and 14 post-CAR delivery. (B) Flow cytometric analysis of CAR transduction efficiency within CD4^+^ and CD8^+^ T cells in unmodified and *FOXP3*-KO T cells of both CAR and bulk TCPs on days 6 and 14 post-CAR delivery. (C) Representative flow cytometry plots showing the T cell memory phenotype distribution based on CCR7 and CD45RA expression in CD4^+^ and CD8^+^ T cells of unmodified and *FOXP3*-KO CAR TCPs on days 6 (top) and 14 (bottom) post-CAR delivery. Naive-like (T_NAIVE-LIKE_: CCR7^+^CD45RA^+^), central memory (T_CM_: CCR7^+^CD45RA^−^), effector memory (T_EM_: CCR7^−^CD45RA^−^), and terminally differentiated effector memory (T_EMRA_: CCR7^−^CD45RA^+^) T cell subsets. (D) Flow cytometric analysis of the T cell memory phenotype distribution based on CCR7 and CD45RA expression in CD4^+^ and CD8^+^ T cells within CAR bulk TCPs (left) and CAR^+^ T cells only within CAR TCPs (middle) and bulk TCPs (right) on days 6 (top) and 14 (bottom) post-CAR delivery. Statics are displayed in the table below. Inter-donor variations were present, and comparisons that did not reach statistical significance are not displayed.

Flow cytometric analysis revealed a consistent shift in CD4^+^/CD8^+^ composition across all TCPs between days 6 and 14 post-CAR delivery, with decreasing CD4^+^ and increasing CD8^+^ T cell frequencies regardless of *FOXP3* status in both CAR-transduced and bulk TCPs (Figure 2A). CAR expression within the CD4^+^ and CD8^+^ subsets of respective *FOXP3*-KO and unmodified CAR TCPs remained stable and comparable from days 6–14 (Figure 2B), with a trend toward higher CAR expression in CD4^+^ compared to CD8^+^ T cells.

Memory phenotype distribution analysis using CD45RA and CCR7 markers classified T cells into four subsets: central memory T cells (T_CM_) (CCR7^+^CD45RA^−^), effector memory T cells (T_EM_) (CCR7^−^CD45RA^−^), terminally differentiated effector memory T cells (T_EMRA_) (CCR7^−^CD45RA^+^), and naïve-like T cells (T_NAIVE-LIKE_) (CCR7^+^CD45RA^+^) (Figure 2C). In the CD4^+^ compartment at day 6, unmodified TCPs predominantly exhibited T_CM_ phenotypes, followed by T_EM_ and T_NAIVE-LIKE_ cells. In contrast, *FOXP3* knockout significantly shifted this distribution in CAR-transduced populations, resulting in fewer T_NAIVE-LIKE_ cells and an increase in T_EM_ cells (Figure 2D, upper panel). By day 14, the CD4^+^ compartment across all conditions shifted predominantly toward T_EM_ and T_EMRA_ phenotypes, with *FOXP3*-KO conditions maintaining a trend toward lower T_NAIVE-LIKE_ percentages in CAR-expressing populations (Figure 2D, lower panel). The CD8^+^ compartment exhibited distinct patterns: at day 6, unmodified TCPs had the highest frequencies of T_NAIVE-LIKE_ cells, with comparable proportions of T_CM_ and T_EMRA_ cells. In *FOXP3*-KO CAR-transduced populations, this distribution shifted markedly: T_EMRA_ cells became more prominent, T_NAIVE-LIKE_ cells were significantly reduced, and T_EM_ cells increased compared to unmodified CAR TCPs (Figure 2D, upper panel). By day 14, all CD8^+^ populations shifted toward T_EMRA_ and T_EM_ phenotypes, with *FOXP3*-KO conditions consistently showing a trend toward decreased T_NAIVE-LIKE_ cell frequencies (Figure 2D, lower panel), although these results showed stronger inter-donor variation and differences were not statistically significant.

### *FOXP3* knockout enhances CAR T cell functionality despite similar exhaustion marker profiles

**Figure 3:**
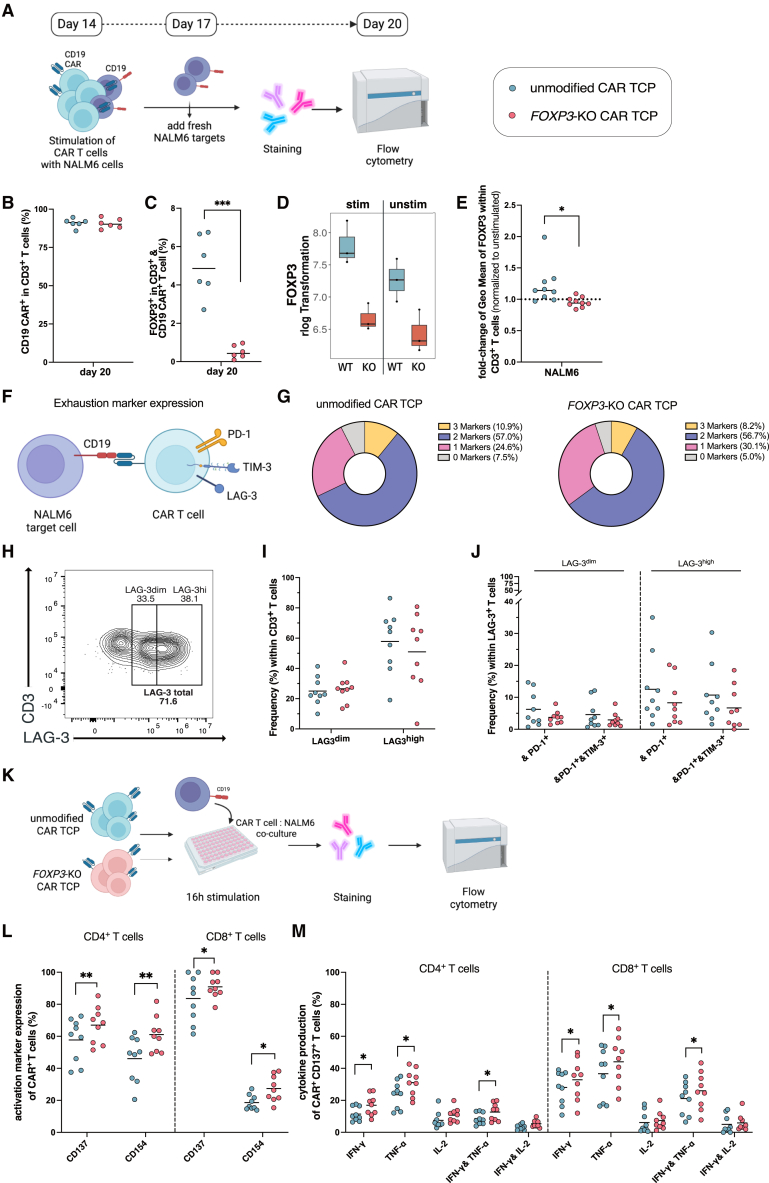
*FOXP3*-KO CAR TCPs display increased effector cytokine production compared to unmodified CAR TCPs despite similar exhaustion marker profiles Data represent mean from *n* = 9 independent donors (one dot in each graph represents one independent donor), unless stated otherwise. ∗*p* < 0.05, ∗∗*p* < 0.01, ∗∗∗*p* < 0.001. (A) Schematic overview of CAR T cell co-culture with CD19-expressing NALM6 target cells. On day 14 post-CAR delivery, unmodified and *FOXP3*-KO CAR TCPs were co-cultured with NALM6 targets. Fresh targets were added every 3 days following flow analysis. Created with BioRender.com. (B and C) Flow cytometric analysis of CAR (B) and FOXP3 (C) expression levels in unmodified and *FOXP3*-KO CAR TCPs after two rounds of repetitive antigen stimulation in CAR-T:NALM6 co-culture (day 20 post-CAR delivery). *n* = 6 independent donors. (D) RNA sequencing analysis of *FOXP3* transcript levels in unmodified and *FOXP3*-KO CAR TCPs upon CAR stimulation or when left unstimulated. *n* = 3 independent donors. (E) Flow cytometric analysis of activation-induced FOXP3 upregulation in unmodified CAR TCPs following short-term overnight NALM6 stimulation (normalized to unstimulated controls). (F) Schematic overview of exhaustion marker expression (LAG-3, PD-1, TIM-3) after CAR T cell and NALM6 target cell engagement. Created with BioRender.com. (G) Mean percentage of CAR T cells expressing zero, one, two, or three exhaustion markers in unmodified (left) versus *FOXP3*-KO (right) CAR TCPs after six rounds of antigen exposure in CAR-T:NALM6 co-culture (inter-donor variability present, data not statistically significant). (H) Representative flow cytometry plot showing LAG-3-expressing T cells within CD3^+^ T cells can be divided into LAG-3^dim^ and LAG-3^high^ populations. (I) Flow cytometric analysis of LAG-3^dim^- and LAG-3^high^-expressing populations within CD3^+^ T cells of unmodified and *FOXP3*-KO CAR TCPs after six rounds of antigen exposure in CAR-T:NALM6 co-culture, (inter-donor variability present, data not statistically significant). (J) Flow cytometric analysis of co-expression patterns of PD-1 alone or PD-1 and TIM-3 together within LAG-3^dim^- and LAG-3^high^-expressing populations after six rounds of antigen exposure in CAR-T:NALM6 co-culture, (inter-donor variability present, data not statistically significant). (K) Schematic overview of the overnight stimulation assay on day 14 post-CAR delivery. Unmodified and *FOXP3*-KO CAR TCPs were co-cultured with NALM6 targets for 16 h in presence of brefeldin A to capture intracellular cytokine production via flow analysis. Created with BioRender.com. (L) Flow cytometric analysis of activation marker expression CD154 and CD137 of CD4^+^ and CD8^+^ CAR^+^ T cells of unmodified and *FOXP3*-KO CAR TCPs (data are background subtracted). (M) Flow cytometric analysis of intracellular effector cytokine production (IFN-γ, TNF-α, and IL-2) of CD4^+^ and CD8^+^ CAR^+^ T cells of unmodified and *FOXP3*-KO CAR TCPs (data are background subtracted).

To mimic persistent antigen exposure seen in clinical settings, we established a sequential antigen stimulation protocol involving repeated co-culture of CAR TCPs with CD19-expressing NALM6 target cells (Figure 3A). Starting on day 14 post-CAR delivery, CAR TCPs were co-cultured at a 3:1 effector-to-target ratio, and fresh NALM6 cells were added after 3 days in the same E:T ratio. Flow cytometric analysis was performed on day 20. Both unmodified and *FOXP3*-KO CAR TCPs demonstrated comparable proliferative response and enriched to ∼90% of T cells expressing the CAR by day 20 of co-culture (Figure 3B). FOXP3 expression remained significantly elevated in unmodified compared to *FOXP3*-KO CAR TCPs following sequential stimulation (Figure 3C), confirming sustained *FOXP3* disruption. These results were validated by RNA sequencing, which showed activation-induced *FOXP3* transcript upregulation in unmodified CAR TCPs (Figure 3D), and by flow cytometry after short-term NALM6 target cell stimulation (Figure 3E).

To assess exhaustion phenotypes, we analyzed expression of three canonical exhaustion markers LAG-3, PD-1, and TIM-3 after six rounds of sequential antigen re-exposure (Figure 3F). Modest differences were observed between groups: unmodified CAR TCPs had a higher frequency of cells co-expressing all three markers (10.9% vs. 8.2%), while *FOXP3*-KO CAR TCPs showed a higher proportion of single-positive cells (30.1% vs. 24.6%); results showed donor-to-donor variation, but differences were not statistically significant (Figure 3G). Further analysis of LAG-3 expression (Figures 3H–3J) revealed that *FOXP3*-KO CAR TCPs had more LAG-3^dim^ and fewer LAG-3^high^ cells (Figure 3I), with reduced co-expression of PD-1 and TIM-3 across conditions (Figure 3J); while these results showed inter-donor variation, differences were not statistically significant. However, RNA sequencing showed comparable upregulation of exhaustion marker and cytokine transcripts after 6-h CAR stimulation in both CAR TCPs across all donors (Figure S1G).

Despite modest exhaustion marker differences, functional analysis revealed substantial enhancements in *FOXP3*-KO CAR TCPs. After 16-h CAR T cell:NALM6 co-culture (Figure 3K), *FOXP3*-KO CAR TCPs demonstrated significantly higher expression of activation markers CD154 (CD40L) and CD137 (4-1BB) in both the CD4^+^ and CD8^+^ subsets (Figure 3L). Effector cytokine production was significantly enhanced for interferon gamma (IFN-γ) and tumor necrosis factor alpha (TNF-α), including increased co-production, in both CD4^+^ and CD8^+^ *FOXP3*-KO CAR-expressing T cells (Figure 3M). IL-2 production and co-production with IFN-γ trended higher in CD4^+^ *FOXP3*-KO CAR-expressing TCPs while remaining comparable in CD8^+^ T cells. Parallel stimulation of CAR-lacking bulk TCPs showed no specific upregulation of activation markers, confirming the antigen specificity of activation marker expression (Figure S1H).

### Cytotoxicity of *FOXP3*-KO CAR TCPs in serial killing assays

Starting on day 14 post-CAR delivery, serial killing assays were used to assess the ability of CAR TCPs to eliminate CD19-expressing targets across multiple rounds of antigen exposure (Figure S1I). Co-cultures of TCPs with GFP-labeled NALM6 targets at distinct effector-to-target ratios (3:1 and 1:1) were monitored with fresh targets added every 3 days over seven consecutive rounds. As expected, CAR-lacking bulk TCPs showed no killing activity, while both CAR TCPs demonstrated cytotoxicity with varying patterns across ratios, donors, and rounds, although differences did not reach statistical significance. At 3:1 ratio, both CAR TCPs showed comparable killing during the first three rounds, with reduced activity in the second round (Figure S1J). From round four, *FOXP3*-KO CAR TCPs exhibited a trend toward better killing capacity. At 1:1 ratio, patterns were more variable (Figure S1K), with *FOXP3*-KO CAR TCPs trending to perform better in rounds two, five, six, and seven, while unmodified CAR TCPs tend to show superior killing in round three.

## Discussion

Despite the clinical success of CAR T cell therapy in hematologic malignancies, its broader application is hindered by challenges such as T cell exhaustion and limited persistence, particularly in solid tumors. Understanding the mechanisms that regulate CAR T cell function is essential for overcoming these barriers and improving therapeutic efficacy.^4^^,^^5^ One factor implicated in T cell dysfunction is the transcription factor FOXP3, best known for its role in Tregs, but also transiently expressed in Teff and tumor-infiltrating lymphocytes (TILs) upon activation. While emerging evidence suggests that FOXP3 may negatively regulate Teff function,^11^^,^^13^ the direct impact of *FOXP3* disruption on CAR T cells remains largely unexplored. This knowledge gap is particularly important given that *FOXP3* knockout in Tregs restores effector cytokine production, highlighting its role in suppressing effector functions.^14^ We therefore aimed to investigate the effects of targeted *FOXP3* knockout on CAR T cell potency and exhaustion profiles. Several recent studies have explored *FOXP3* biology in human-derived CAR T cells, most notably in the CAR-Treg context, where supraphysiological FOXP3 expression enhanced stability and function^17^ and where OX40L-CAR-Tregs, driven by a FOXP3 promoter, controlled alloreactivity.^18^ Notably, a recent study by Niu et al.^19^ investigated FOXP3 overexpression in human CAR T cells, which enhanced their metabolic fitness and persistence, and validated these effects *in vivo* in a humanized immune system mouse model.^19^ In comparison to these FOXP3 overexpression studies (largely in Tregs or in human effector CAR T cells), our work examines *FOXP3* knockout in conventional effector human CAR T cell products generated under clinical manufacturing conditions, focusing on cytokine production and cytotoxicity. These complementary approaches underscore the complexity of FOXP3 biology and highlight the need to understand both gain- and loss-of-function effects across different T cell therapy platforms.

We developed a protocol combining lentiviral CAR delivery with CRISPR-Cas9-mediated *FOXP3* knockout, achieving efficient gene disruption without compromising T cell expansion. This approach is consistent with emerging CRISPR-Cas9 strategies for enhancing T cell therapies.^20^^,^^21^^,^^22^ *FOXP3* knockout did not affect CAR T cell manufacturing parameters, suggesting compatibility with established protocols. However, *FOXP3*-KO CAR TCPs consistently showed reduced CD25 (IL-2Rα) expression, consistent with FOXP3’s role as a direct transcriptional regulator of CD25.^23^ This downregulation may impair IL-2 responsiveness and limit *in vivo* expansion or persistence, particularly in cytokine-limited environments, a trade-off that warrants consideration in clinical applications. This finding further supports the notion that FOXP3 retains regulatory control during T cell activation, potentially influencing IL-2 signaling, which is critical for T cell function.^24^ We generated *FOXP3*-KO CAR TCPs targeting CD19 and compared their functional capacity and exhaustion profiles to unmodified CAR TCPs following repetitive antigen exposure. Our results showed that while *FOXP3*-KO CAR TCPs exhibited similar exhaustion marker expression (LAG-3, PD-1, and TIM-3) compared to unmodified CAR TCPs, they demonstrated a trend toward enhanced effector function, including increased cytokine production and prolonged cytotoxic activity. These findings suggest that FOXP3 may function as a negative regulator of CAR T cell activity and that its disruption may improve CAR T cell therapeutic efficacy.^25^ The upregulation of FOXP3 in unmodified CAR TCPs upon antigen stimulation suggests that persistent signaling may induce a regulatory-like state, potentially limiting sustained effector function. This aligns with prior studies showing that transient FOXP3 expression in Teff is associated with reduced cytokine production and impaired cytotoxicity.^11^^,^^12^^,^^13^ The enhanced functional capacity of *FOXP3*-KO CAR TCPs, despite similar exhaustion marker expression, suggests that FOXP3 influences effector function through mechanisms distinct from classical exhaustion pathways.^10^^,^^11^^,^^13^^,^^26^ The phenotypic shift in *FOXP3*-KO CAR TCPs toward more differentiated effector memory subsets, particularly evident in CAR-expressing cells, suggests accelerated differentiation resulting from a synergy between CAR signaling and FOXP3-dependent differentiation pathways. This is further supported by the reduction in T_NAIVE-LIKE_ populations, indicating a progression toward more effector-driven phenotypes.^16^ However, this skewing may also reflect early terminal differentiation or limited stemness potential. Although no impairment was evident *in vitro*, reduced frequencies of naïve-like or central memory subsets may impact persistence *in vivo*, particularly in settings requiring long-term tumor control. While highly differentiated T cells generally exhibit reduced persistence, certain terminally differentiated effector memory subsets may still retain the ability to proliferate and produce cytokines. This suggests that *FOXP3* knockout could enhance CAR T cell function without necessarily compromising long-term persistence.^27^^,^^28^^,^^29^

FOXP3 interacts with key transcription factors such as nuclear factor of activated T cells (NFATs) and nuclear factor κB (NF-κB), which are central to T cell activation and cytokine production.^26^^,^^30^^,^^31^ Its ability to modulate effector T cell programs through these interactions may help explain why FOXP3-KO CAR TCPs exhibited enhanced cytokine production despite minimal changes in classical exhaustion marker expression. These observations suggest that FOXP3 does not directly induce T cell exhaustion but instead modulates CAR T cell function through a combination of transcriptional and post-transcriptional mechanisms. For instance, the interaction between FOXP3 and RELA, a subunit of NF-κB, implies that FOXP3 knockout could enhance NF-κB-driven transcription, potentially contributing to sustained T cell activation and improved cytotoxicity. However, RNA sequencing revealed no major differences in exhaustion marker expression or cytokine transcript levels between unmodified and FOXP3-KO CAR TCPs. This raises the possibility that FOXP3 may also act through post-transcriptional processes, such as mRNA stability or translation efficiency, rather than by altering gene transcription alone.^32^ FOXP3 has also been implicated in T cell metabolic regulation, particularly in Tregs. While not directly assessed here, *FOXP3* knockout could plausibly influence glycolytic activity or mitochondrial function, contributing to the observed functional shifts.^19^ These possibilities warrant further mechanistic investigation. Furthermore, the absence of substantial epigenetic changes following *FOXP3* knockout, despite its known role in epigenetic reprogramming in Tregs,^7^^,^^33^ suggests that transient FOXP3 expression in conventional T cells may not induce stable chromatin remodeling. Instead, FOXP3 likely exerts its regulatory effects in CAR T cells primarily through dynamic protein-protein interactions. While no enduring epigenetic reprogramming was detected, FOXP3 may still influence T cell dysfunction through more transient or non-canonical mechanisms, including modulation of chromatin accessibility or non-coding RNAs.

Several limitations must be considered, including the *in vitro* nature of our study, which may not fully replicate the clinical microenvironment, as well as donor variability. It remains debatable whether the effects of *FOXP3* disruption observed here are universally beneficial for therapeutic CAR T cells, suggesting value in exploring *FOXP3* knockout in combination with other gene modifications to optimize TCP potency. While our study focused on CD19 CAR T cells, *FOXP3* modulation may also benefit other engineered T cell therapies, including TCR-transduced or solid-tumor-directed products. Investigating *FOXP3* knockout in diverse antigen-specific and microenvironmental contexts will help assess broader applicability. Future studies should explore the post-transcriptional mechanisms through which FOXP3 modulates CAR T cell function, given the differences at the protein level despite minimal transcriptional changes.^11^^,^^13^^,^^14^ Additionally, investigating the differential impact of *FOXP3* loss in naive versus memory T cells could yield important insights, as memory T cells primed for defined antigens may particularly benefit from *FOXP3* disruption, a strategy potentially valuable in settings of chronic infection and cancer. Further investigations into the effects of *FOXP3* knockout in CAR T cells concerning exhaustion-independent pathways or metabolic regulators could be insightful to improve CAR T cell efficacy. *In vivo* studies should follow validation in diverse donor populations and tumor models. Importantly, the observed functional improvements were modest and donor-variable, emphasizing the exploratory nature of this study. Our findings are best viewed as hypothesis-generating and supportive of more mechanistic investigations into the role of FOXP3 in T cell biology.

In conclusion, CRISPR-Cas9-mediated *FOXP3* knockout in CAR TCPs enhances their functional capacity without compromising expansion or significantly altering exhaustion marker expression. Our study provides the first investigation into *FOXP3* knockout in therapeutic T cells, offering evidence that FOXP3 may serve as a negative regulator of effector function, particularly upon chronic antigen exposure. These findings highlight FOXP3 as a potential target for improving CAR T cell therapies, though further investigation is needed to fully understand its regulatory mechanisms and therapeutic potential. In particular, *FOXP3* disruption may be most beneficial in disease settings characterized by persistent antigen exposure or suppressive environments such as solid tumors or chronic viral infections where sustained effector function is critical.

## Materials and methods

### Blood sampling

The study was approved by the Charité-Universitätsmedizin Berlin Ethics Committee, and peripheral blood was obtained from healthy donors, who had given their written informed consent (Ethics Committee of the Charité approval EA4/091/19).

### Generation of CAR T cells

CD3^+^ T cells were isolated from healthy donor PBMCs via Biocoll (Biochrom) gradient centrifugation and magnetic separation (CD3 MicroBeads, Miltenyi Biotec). Cells were cultured in a 1:1 mixture of Click’s medium and Advanced RPMI (Gibco) supplemented with 10% FCS (PAA), 1% GlutaMAX (Gibco) and 10 ng/mL rhIL-7 and rhIL-15 (both CellGenix) in humidified incubators at 37°C, 5% CO_2_. T cells were activated using plate-bound anti-CD3 (1 μg/mL, Okt3, eBioscience) and anti-CD28 (1 μg/mL, BioLegend) antibodies. Lentiviral transduction for CD19 CAR delivery (lentiviral particles, containing a human Fc region, were produced in-house as previously described using transient transfection of 293T cells^16^^,^^34^^,^^35^) was performed 24 h post-T cell activation at MOI of 1 using protamine sulfate (0.1 mg/mL, Sigma-Aldrich) as facilitator, followed by centrifugation (800×g, 30 min, 30°C) and subsequent culture with 1:1 splitting upon reaching confluence.

### Knockout procedure

On day 3 post-T cell isolation, 1 × 10^6^ T cells were electroporated using P3 Primary Cell 4D-Nucleofector X Kit S and the Amaxa-Nucleofector-4D (Lonza, program EH-115), with pre-complexed RNP containing two *FOXP3*-specific sgRNAs (sgRNA#1: GGGCCATCGCAGCTCAAAGT; sgRNA#2: GGGGCCGAGATCTTCGAGGC, Synthego Corporation), poly(L-glutamic acid) (100 μg/μL, Sigma) and recombinant Alt-R Hifi *Streptococcus pyogenes* Cas9 protein V3 (Integrated DNA Technologies) in a 0.96:1:0.8 volume ratio. After electroporation, cells were recovered with pre-warmed T cell medium and incubated at 37°C for 15 min before transfer to 96-well round-bottom plates at 0.5×10^6^ cells/well. Unmodified CAR T cells and electroporated cells without additives served as controls.

### Knockout efficiency analysis

Analysis of on-target editing was performed from isolated DNA (Zymo Research) of day 6 and day 14 cell samples. The *FOXP3* locus was amplified using KAPA HiFi HotStart ReadyMix (Roche) and the following primer pairs—AAGCAGCGGACACTCAATGA (F) and GCAGGCAAGACAGTGGAAAC (R)—with the following touchdown-PCR program in an automated thermocycler: (1) 95°C, 3 min; (2) 98°C, 20 s; (3) 62°C, 15 s; (4) 72°C, 10 s; (5) 72°C, 20 s; and (6) 12°C. PCR products were purified using DNA purification and enrichment kit (Zymo Research) prior Sanger sequencing with primer F/F′ by LGC Genomics GmbH. Editing frequencies were calculated using the Inference of CRISPR Edits (ICE) algorithm (Synthego Corporation).

### Phenotypic and functional assays assessed by flow cytometry

To assess the *FOXP3* knockout efficiency and CAR frequency of TCPs, we stained T cells using antibodies (all from BioLegend, unless stated otherwise) and the FoxP3/Transcription Factor Staining Buffer Set (eBioscience). Staining of CD19 CAR was performed using PE-Labeled Human CD19 Protein, His Tag (ACROBiosystems), while staining of remaining markers was carried out using fluorophore-conjugated human anti-CD3 (OKT3), -CD4 (SK3), -CD8 (RPA-T8), -FoxP3 (259D), -CCR7 (G043H7), and CD45RA (HI100) antibodies. LIVE/DEAD Fixable Blue Dead Cell Stain (L/D; Invitrogen) was used to identify living cells.

For assessment of CD19 CAR T cell cytokine production/activation, CD19-expressing NALM6 cells were used as antigen-presenting cells at a 3:1 (E:T) ratio for a 16-h antigen-specific stimulation. Unstimulated controls, lacking NALM6 targets, served as negative control, while phorbol myristate acetate (PMA)- and ionomycin-stimulated cells served as positive control. Intracellular cytokine production was captured by addition of 2 μg/mL of Brefeldin A (Sigma-Aldrich) after 1 h of stimulation and following 16 h total stimulation, T cells were stained using antibodies (all from BioLegend, unless stated otherwise) and the FoxP3/Transcription Factor Staining Buffer Set (eBioscience). Staining was performed using fluorophore-conjugated human anti-CD3 (OKT3), -CD4 (SK3), -CD8 (RPA-T8), -IFN-γ (4S.B3), -TNF-α (MAb11), -IL-2 (MQ1-17H12), -CD137 (4B4-1), and -CD154(24–31) antibodies. LIVE/DEAD Fixable Blue Dead Cell Stain (L/D; Invitrogen) was used to identify living cells.

For assessment of exhaustive phenotype of CAR T cells following repetitive antigen-exposure, we co-cultured CD19 CAR TCPs with CD19-expressing NALM6 cells at a 3:1 (E:T) ratio for a total of 21 days. Fresh target cells were added every 3 days at a 3:1 ratio. To assess exhaustion marker expression, T cells were stained using antibodies (all from BioLegend, unless stated otherwise). Staining was performed using fluorophore-conjugated human anti-CD3 (OKT3), -CD4 (SK3), -CD8 (RPA-T8), -LAG-3 (3DS223H, Invitrogen), -PD-1 (EH12.2H7), and -TIM-3 (F38-2E2) antibodies. LIVE/DEAD Fixable Blue Dead Cell Stain (L/D; Invitrogen) was used to identify living cells.

All flow cytometry samples were analyzed using a CytoFLEX flow cytometer (Beckman Coulter) and FlowJo-10 software (Tree Star).

### Serial killing assay

For assessment of serial killing capacity of CAR T cells following repetitive antigen exposure, we co-cultured CD19 CAR TCPs with CD19-expressing GFP-labeled NALM6 cells at distinct effector-to-target ratios (3:1 and 1:1) for a total of seven consecutive rounds. Fresh target cells were added every 3 days. GFP-positive target cells were imaged every 4 hours using the Incucyte SX5 device (Sartorius) in 96-well clear-bottom imaging plates (PhenoPlate, Revvity). The number of GFP-positive target cells over time was quantified, serving as a proxy for viable target cells. Simultaneous co-cultures with bulk TCPs served as control. Data were normalized to target cell count at the first time point of each round, and killing percentage and corresponding area under the curve were calculated for each round. All downstream analysis was performed in R using the packages tidyverse,^36^ ggplot2,^37^ and janitor.^38^

### RNA bulk sequencing

To assess transcriptional changes in the RNA profile of unmodified and *FOXP3*-KO TCPs, CAR TCPs were stimulated in a 96-well flat-bottom plate coated with 10 ng/mL anti-human immunoglobulin G (IgG), Fc γ Fragment (Jackson ImmunoResearch) for 6 h following RNA isolation using RNeasy Micro Kit (Qiagen). Illumina libraries were generated using SMART-Seq v4 Ultra Low Input RNA Kit (Takara Clontech) and Nextera XT DNA Sample Preparation Kit (Illumina), with up to 10 ng of purified complementary DNA (cDNA). The quality of synthesized cDNA was checked using Bioanalyzer (Agilent), and sequencing was performed using Illumina NextSeq2000 device. Raw sequence reads were mapped to hg38 gene using Tophat2^39^ and Bowtie2^40^ with very sensitive settings. Read counts were determined with featureCounts.^41^ Further analysis was done with R using the default settings of the deseq2 package.^42^ Heatmap representing log2 of normalized transcript counts were plotted using *pheatmap* package.^43^

### DNA methylation profiling

DNA methylation analysis was performed on isolated DNA (Zymo Research) of day 16 TCP samples. Bisulfite conversion of samples was performed using the EZ-DNA methylation Gold Kit (Zymo Research). DNA methylation was assessed using the Infinium MethylationEPIC Kit (Illumina EPIC-8 BeadChip) following manufacturer’s instructions and as previously reported.^44^ The raw intensity data files (IDAT) underwent preprocessing utilizing minfi version 1.42.0^45^ through quantile normalization. Probes were filtered based on criteria such as failing to meet the detection *p* value threshold against the background (*p* < 0.01), being identified as cross-reactive, revisions in Illumina’s manifest (Infinium MethylationEPIC v1.0 13.03.2020), or residing at an SNP locus, employing the minfi function dropLociWithSnps. R statistical software (Version >4.0) was used for all further data analysis.

### Statistical analysis

*p* values were determined by tests for normal distribution (Shapiro-Wilk and Kolmogorov-Smirnov tests), followed by one-way ANOVA (normally distributed datasets) or Friedman test (not normally distributed datasets) and paired t tests (normally distributed datasets) or Wilcoxon matched-pairs signed rank tests (not normally distributed datasets) as posttests.

## Data availability

All data needed to evaluate the conclusions in the paper are present in the paper and/or the supplemental information. There are no restrictions on the use of materials. All data generated in this study are available as source data or from the corresponding author on reasonable request.

## Acknowledgments

We thank all voluntary blood donors for their generous contributions. We also thank Rebecca Friedrich, Onur Akça, Clemens Franke, Anne Schulze, and Shirley Lugo for their technical assistance with PCRs, Sanger sequencing preparation for KO efficiency assessments, and generation of DNA methylation data. The study was supported in parts by the 10.13039/501100002347German Federal Ministry of Education and Research (BIH Center for Regenerative Therapies, 13353, Berlin to M.S.-H. and J.K.P.). This project has received funding from the European Union’s horizon 2020 research and innovation program under grant agreement no. 825392 (Reshape, www.reshape-h2020.eu to M.S.H. and J.K.P.). The funders had no role in study design, data collection and analysis, decision to publish, or preparation of the manuscript.

## Author contributions

L.P. designed the study, planned and performed experiments, composed the figures, analyzed results, interpreted the data, and wrote the manuscript. F.A.Y. and N.H.D. performed experiments. M.F.S., S.P., and S.S. acquired, analyzed, and interpreted data. A.K., J.K., and D.L.W. provided the lentiviral CAR construct and interpreted data. M.-F.M. and F.Heinrich. analyzed and interpreted the RNA sequencing data and provided the necessary infrastructure. F.Hamm. and J.K.P. analyzed and interpreted the DNA methylation data and provided the necessary infrastructure. P.R. provided infrastructure and interpreted data. M.S.-H. led the project, conceptualized and designed the research, interpreted the data, and wrote the manuscript. All authors discussed, commented on, and approved the manuscript in its final form.

## Declaration of interests

All authors declare no competing interests.

## Declaration of generative AI and AI-assisted technologies in the writing process

During the preparation of this work, the author(s) used Claude by Anthropic (claude.ai) in order to restructure and refine sentences and paragraphs for clarity and flow. After using this tool/service, the authors reviewed and edited the content as needed and take full responsibility for the content of the publication.

## Supplemental information

Document S1. Figure S1

Document S2. Article plus supplemental information
