# Supplementary material for: Effects of CRISPR-Cas9-mediated FOXP3 knockout on CAR T cell potency
Source: Mol Ther Methods Clin Dev. 2025 Aug 21;33(3):101570. doi: 10.1016/j.omtm.2025.101570 (PMC12433514; doi:10.1016/j.omtm.2025.101570)
Supplement: Document S1. Figure S1 [file mmc1.pdf]

OMTM, Volume *33*

Supplemental information

Effects of CRISPR-Cas9-mediated *FOXP3* knockout on CAR T cell potency

Lena Peter, Martí Farrera-Sal, Ferhat Ali Yaman, Nils Henrik Dempewolf, Samira Picht, Sarah Schulenberg, Jonas Kath, Frederik Hamm, Frederik Heinrich, Dimitrios L. Wagner, Mir-Farzin Mashreghi, Annette Künkele, Petra Reinke, Julia K. Polánsky, and Michael Schmueck-Henneresse

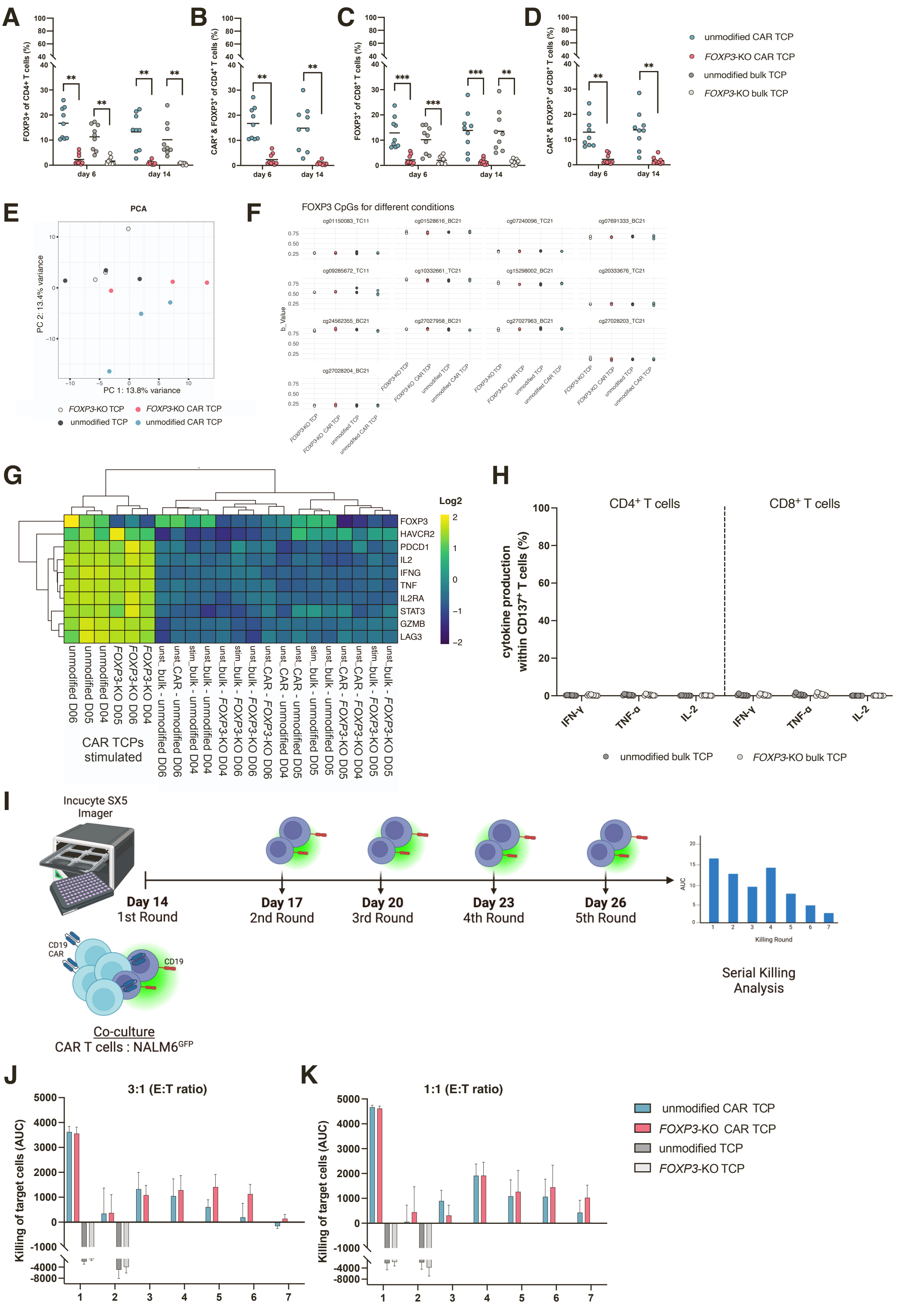

Figure S1: FOXP3 knockout validation, serial killing capacity and epigenetic analysis of unmodified and FOXP3-KO CAR TCPs.

Data represent mean from n=9 independent donors (one dot in each graph represent one independent donor), unless stated otherwise. **p<0.01, ***p<0.001

(A) Flow cytometric analysis of FOXP3 expression levels in $CD4^+$ CAR and bulk TCPs at day 6 and 14 post-CAR delivery.

(B) Flow cytometric analysis of FOXP3 expression levels in $CD4^+$ CAR^+ TCPs at day 6 and 14 post-CAR delivery.

(C) Flow cytometric analysis of FOXP3 expression levels in $CD8^+$ CAR and bulk TCPs at day 6 and 14 post-CAR delivery.

(D) Flow cytometric analysis of FOXP3 expression levels in $CD8^+$ CAR^+ TCPs at day 6 and 14 post-CAR delivery.

(E) Principal component analysis (PCA) of DNA methylation profiles (based on EPIC arrays) in unmodified and *FOXP3*-KO TCPs with or without CAR transduction. n=3 independent donors.

(F) DNA methylation levels of DNA methylation sites within the *FOXP3* gene. n=3 independent donors.

(G) Heatmap of RNA sequencing analysis of unmodified and *FOXP3*-KO CAR and bulk TCPs (unstimulated and CAR-stimulated). n=3 independent donors, Log 2 of normalized transcript count are plotted.

(H) Flow cytometric analysis of intracellular effector cytokine production (IFNγ, TNFα and IL-2) within $CD4^+$ and $CD8^+$ T cells of unmodified and *FOXP3*-KO bulk TCPs (data is background subtracted).

(I) Schematic overview of serial killing assay by co-culturing CAR TCPs with GFP-labeled NALM6 target cells at distinct effector-to-target ratios (3:1 and 1:1), with target cell elimination monitored by Incucyte SX5 imaging and fresh targets added every 3 days.

(J, K) Area under the curve (AUC) analysis of target cell elimination across seven consecutive killing rounds at 3:1 (J) and 1:1 (K) effector-to-target ratios. Mean of n=6 independent donors, ± SEM, inter-donor variability present and data not statistically significant.
